# Biochemical testing for the diagnosis of Wilson's disease: A systematic review

**DOI:** 10.1002/jcla.24191

**Published:** 2021-12-23

**Authors:** Hafiz Muhammad Salman, Mahwish Amin, Javaria Syed, Zouina Sarfraz, Azza Sarfraz, Muzna Sarfraz, Maria Jose Farfán Bajaña, Miguel Felix, Ivan Cherrez‐Ojeda

**Affiliations:** ^1^ Services Institute of Medical Sciences Lahore Pakistan; ^2^ Sir Ganga Ram Hospital Lahore, Punjab Pakistan; ^3^ Sargodha Medical College Sargodha Pakistan; ^4^ Fatima Jinnah Medical University Lahore Pakistan; ^5^ The Aga Khan University Karachi Pakistan; ^6^ King Edward Medical University Lahore Pakistan; ^7^ Universidad Espíritu Santo Samborondón Ecuador; ^8^ Respiralab, Respiralab Research Group Guayaquil Ecuador

**Keywords:** ceruloplasmin, hepatic copper, hepatolenticular degeneration, liver copper, urinary copper, Wilson's disease

## Abstract

**Background:**

Wilson's disease (WD) is a rare inherited disorder that leads to copper accumulation in the liver, brain, and other organs. WD is prevalent worldwide, with an occurrence of 1 per 30,000 live births. Currently, there is no gold standard diagnostic test for WD. The objective of this systematic review is to determine the diagnostic accuracy for WD of three biochemical tests, namely hepatic copper, 24‐hour urinary copper, and ceruloplasmin using the Leipzig criteria.

**Methods:**

Adhering to PRISMA guidelines, databases including PubMed/MEDLINE, CINAHL Plus, Web of Science, and Cochrane were searched. Studies that comprised of confirmed or suspected WD along with normal populations were included with adult and pediatric group. The sensitivity, specificity, negative predictive value and positive predictive value were computed using RevMan 5.4.

**Results:**

Nine studies were included. The best practice evidence for 24‐hour urinary copper test ranged from a cutoff value of 0.64–1.6 μmol/24 h (*N* = 268; sensitivity = 75.6%, specificity = 98.3%). Hepatic copper test was optimally cutoff based on the ROC curve analysis at 1.2 μmol/g yielding a power of 96.4% sensitivity and 95.4% specificity (*N* = 1,150); however, the tried and tested 4 μmol/g cutoff, with 99.4% sensitivity and 96.1% specificity, is more widely accepted. The ceruloplasmin test cutoff value was found to be ranging from 0.14 to 0.2 g/L (*N* = 4,281; sensitivity = 77.1%–99%, specificity = 55.9%–82.8%).

**Conclusion:**

This paper provides a large‐scale analysis of current evidence pertaining to the biochemical diagnosis of WD employing the Leipzig criteria. The laboratory values are typically based on specific subgroups based on age, ethnicity, and clinical subgroups. The findings of this systematic review must be used with caution, given the over‐ or under‐estimation of the index tests.

## Graphical abstract

Wilson's disease bears a burden on the adult and pediatric population worldwide, with a prevalence of 1 per 30,000 live births. There is no gold standard test for the disease. This systematic review analyzes large‐scale data to find the best practice evidence for the diagnosis of Wilson's disease in symbiosis with the Leipzig criteria.

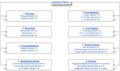

## 1 INTRODUCTION

Wilson's disease (WD) was first described in 1912 by Samuel Wilson as an autosomal recessive metabolic disorder occurring due to mutations of the ATP7B gene.^1^ It is a rare inherited disorder that leads to copper accumulation in the liver, brain, and other vital organs.^2^ WD is found worldwide, with an estimated prevalence of 1 per 30,000 live births across populations,^3^ although the data obtained by molecular sequencing from the United Kingdom suggest higher prevalence of 1 per 7,021 live births.^4^ A large proportion of patients are diagnosed between the ages of 5 and 35 years, but it may affect the older population as well.^1^, ^2^ At present, there is no gold standard diagnostic test for Wilson's disease, where only the measurements of liver copper content are being used to improve the diagnostic accuracy.^5^ Owing to the nonspecific clinical features of Wilson's disease, the battery of laboratory and clinical tests for diagnosis is oftentimes delayed.^6^ Ultimately, this may affect the clinical outcomes and has implications for members in the family tree when considering late or missed diagnosis earlier during the disease course.^7^, ^8^ With the Leipzig criteria, the shortcoming of no gold standard diagnostic test may be overcome by promoting the standardization of diagnosis and treatment of disease.^9^

The objective of this systematic review is to determine the diagnostic accuracy, that is, sensitivity and specificity of biochemical tests, including hepatic copper, 24‐hour urinary copper content, and ceruloplasmin. These laboratory markers are ideally tested in suspected patient and control groups. With inconsistent gold standard testing for the diagnosis of Wilson's disease, the Leipzig criteria are used as the standard for this investigation. The key purpose is to scale the benefits of these tests for patients under an index of suspicion or whether the test should be used at large.

## 2 MATERIALS AND METHODS

**FIGURE 1:**
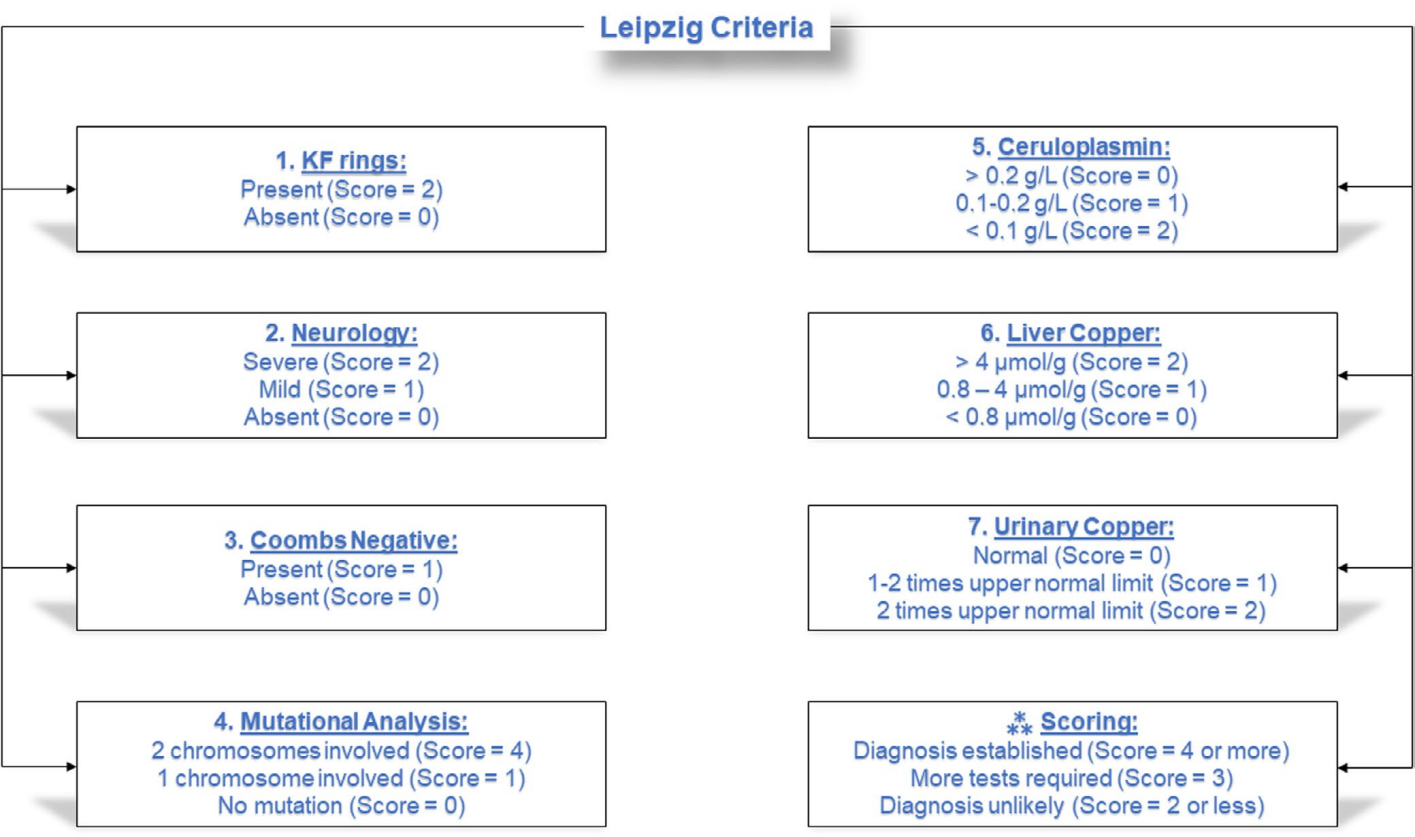
Diagnostic algorithm for Wilson's disease based on the Leipzig score

In accordance with the Preferred Reporting Items for Systematic Reviews and Meta‐Analyses (PRISMA) Statement 2020,^10^ observational studies (retrospective/prospective cohorts and case controls) that tested the diagnostic accuracy of any or all three index tests in the context of diagnosing WD were included. Databases including the following were searched: PubMed/MEDLINE, CINAHL Plus, Web of Science, and Cochrane. The date of the last database search was October 20, 2021. There were no language restrictions. A combination of the following keywords was used: Wilson's disease, Wilson's disease, ceruloplasmin, urinary copper, hepatic copper, liver copper, and hepatolenticular degeneration. The studies included participants with confirmed or suspected WD and also comprised of normal population and heterozygotes in cases where genetic testing was used. In case the study evaluated the index test in the normal population with the Leipzig criteria but did not include a WD comparator group, it was omitted. The target population was the pediatric and adult population with suspected WD as assessed by the Leipzig criteria, which was essential for inclusion. Studies that did not use the Leipzig criteria and did not define WD were excluded. The three index tests were ceruloplasmin, liver copper content, and urinary copper that were evaluated for diagnosing WD. The cutoff thresholds for each of the index test are provided in Figure 1. The clinical reference standard to the diagnosis of WD is outlined in the Leipzig criteria below.

Two pairs of authors (AS‐ZS and HMS‐JS) independently extracted data onto a google spreadsheet, and any discrepancies were resolved by active discussion. The data were entered as author and year, participant and methodology, clinical characteristics, index test details, the reference standard used in the study, and biases of the included studies. The sensitivity, specificity, positive predictive value (PPV), and negative predictive value (NPV) were calculated using ReviewManger 5.4 (RevMan 5.4, Cochrane), which are tabulated for all three index tests in Tables 1, 2, 3. The data were entered into the software as the number of TP/ participants with WD (TP + FN). The higher the sensitivity for a particular cutoff, the better the test was considered in correctly identifying individuals with WD. The specificity was calculated as the number of TN/ participants without WD (TN + FP). The higher the specificity of the cutoff value, the better the diagnostic test in identifying individuals who do not have WD. PPV is the number of TP/ numbers of TP + FP. PPV measures the individuals with positive tests who have WD. NPV is the number of TN/numbers of TN +FN. NPV measures the number of people with a negative test result that do not have WD. The methodology encompassed a diagnostic analytical technique to determine the diagnostic cutoffs of the biochemical tests.

**TABLE 1:** Ceruloplasmin diagnostic accuracy (sensitivity, specificity, PPV, and NPV using 95% CI)

Author (Year)	Threshold	Sensitivity vs. Specificity (95% CI)	PPV vs. NPV (95% CI)
Mak et al. (2008)^15^	0.1 g/L	78.9% (CI: 66.1–88.6) vs. 100% (CI: 97.3–100)	100% (CI: NE) vs. 91.9% (CI: 87.3–94.9)
Nicastro et al. (2010)^11^	0.1 g/L	65% (CI: 66.1–88.6) vs. 96.6% (CI: 88.1–99.6)	92.9% (CI: 76.6–98.1) vs. 80% (CI: 72.3–86)
Sezer et al. (2014)^12^	0.2 g/L	77.1% (CI: 59.9–89.6) vs. 65.9% (CI: 49.4–79.9)	65.9% (CI: 54.9–75.4) vs. 77.1% (CI: 63.9–86.6)
Xu et al. (2018)^13^	0.2 g/L	99% (CI: 97.1–99.8) vs. 80.9% (CI: 79.6–82.2)	29.1% (CI: 27.8–30.5) vs. 99.9% (CI: 99.7–100)
Merle et al. (2009)^14^	0.19 g/L	93.6% (CI: 87.3–97.4) vs. 58.8% (CI: 44.2–72.4)	83.1% (CI: 77.9–87.2) vs. 81% (CI: 66.9–90.1)

Abbreviations: CI, confidence interval; NE, not estimated; NPV, negative predictive value; PPV, positive predictive value.

**TABLE 2:** 24‐hour urinary copper test diagnostic accuracy (sensitivity, specificity, PPV, and NPV using 95% CI)

Author (Year)	Threshold	Sensitivity vs. Specificity (95% CI)	PPV vs. NPV (95% CI)
Nicastro et al. (2010)^11^	0.64 μmol/24 h	78.9% (CI: 62.7–90.5) vs. 87.9% (CI: 76.7.1–95)	81.1% (CI: 67.7–89.7) vs. 86.4% (CI: 77.4–92.2)
Nicastro et al. (2010a)^11^	1.6 μmol/24 h	65.8% (CI: 48.7–80.4) vs. 98.3 (CI: 90.8–100)	96.2 (CI: 77.9–99.4) vs. 81.4 (CI: 73.8–87.2)
Lu et al. (2010)^16^	1.6 μmol/24 h	50% (CI: 29.9–70.1) vs. 97.1% (CI: 89.8–99.6)	86.7% (CI: 61.1–96.4) vs. 83.5% (CI: 77.5–88.2)
Sezer et al. (2014)^12^	1.6 μmol/24 h	80% (CI: 63.1–91.6) vs. 75.6% (CI: 59.7–87.6)	73.7% (CI: 61.4–83.1) vs. 81.6% (CI: 69.1–86.4)

Abbreviations: CI, confidence interval; NPV, negative predictive value; PPV, positive predictive value.

**TABLE 3:** Hepatic copper test diagnostic accuracy (sensitivity, specificity, PPV, and NPV using 95% CI)

Author (Year)	Threshold	Sensitivity vs. Specificity (95% CI)	PPV vs. NPV (95% CI)
Ferenci et al. (2005)^17^	>4 μmol/g	88.3% (CI: 75.2–89.7) vs. 98.6% (CI: 96.1–99.7)	96.9% (CI: 91.1–99) vs. 91.9% (CI: 88.3–94.5)
Ferenci et al. (2005a)^17^	>1.2 μmol/g	96.5% (CI: 91.3–99) vs. 95.4% (CI: 91.8–97.8)	91.7% (CI: 85.7–95.3) vs. 98.1% (CI: 95.2–99.3)
Nicastro et al. (2010)^11^	>4 μmol/g	93.3% (CI = 77.9–99.2) vs. 52.2% (CI = 37–67.1)	56% (CI: 48.1–63.6) vs. 92.3% (CI: 75.4–97.9)
Sezer et al. (2014)^12^	>4 μmol/g	65.7% (CI = 47.8–80.9) vs. 75.6% (CI = 59.7–87.6)	69.7% (CI: 56.1–80.6) vs. 72.1% (CI: 61.3–80.8)
Yang et al. (2015)^18^	>4 μmol/g	94.4% (CI = 89.9–97.3) vs. 96.8% (CI = 94.7–98.2)	91.8% (CI: 87.2–94.9) vs. 97.8% (CI: 96.1–97.5)

Abbreviations: CI, confidence interval; NPV, negative predictive value; PPV, positive predictive value.

## 3 RESULTS

**FIGURE 2:**
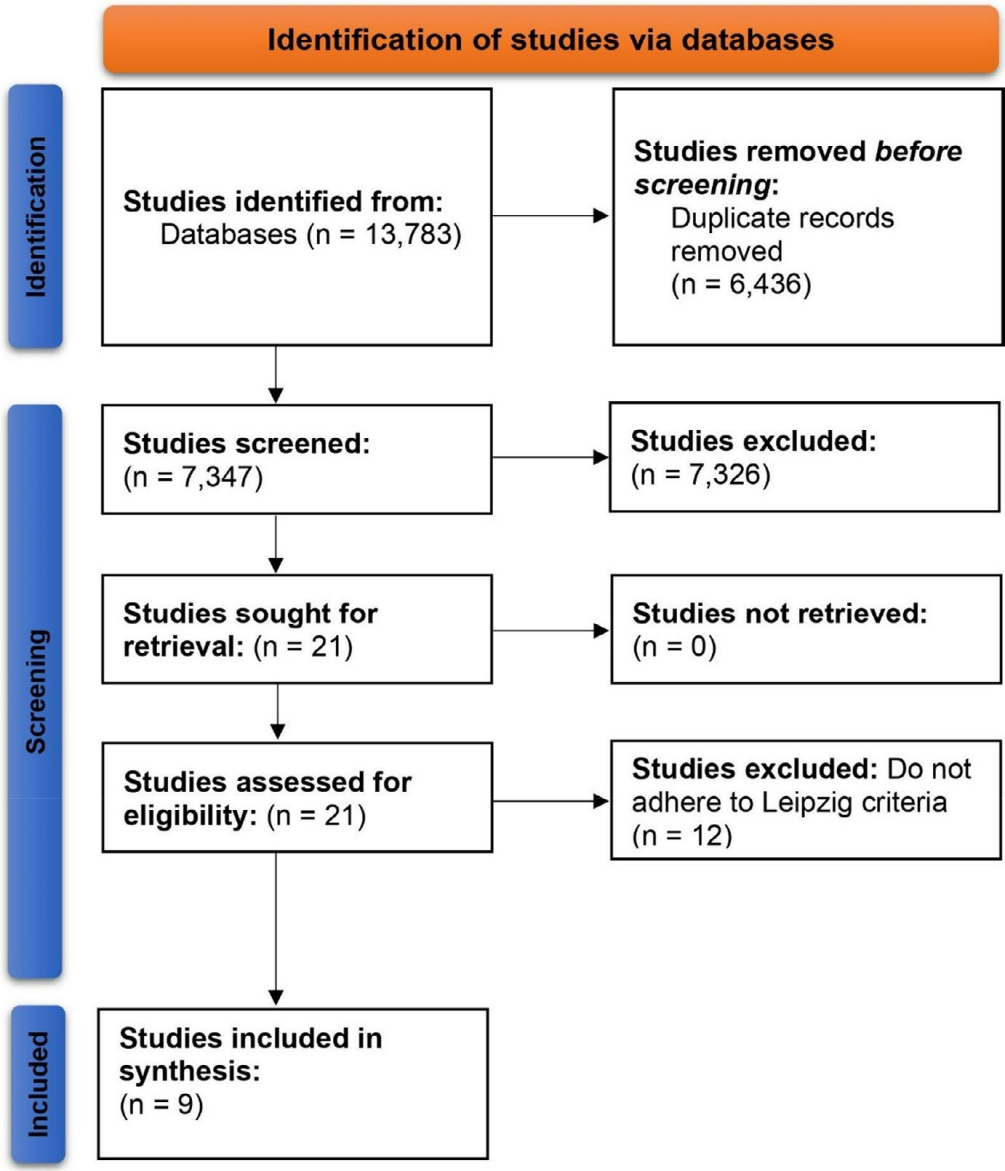
PRISMA flowchart

The PRISMA flowchart is depicted in Figure 2. In total, 13,783 studies were identified from the enlisted databases. On removing 6,436 duplicates, 7,347 studies were screened. Overall, 21 studies were retrieved and thereby assessed for eligibility. Finally, 9 studies were included in this analysis.

### 3.1 Characteristics of included studies

The characteristics of the included studies are listed in Table 4. Only 5 of the 9 studies provided data for ceruloplasmin. Overall, while 6 of the 9 studies were case controls, with one each being a cross‐sectional, a prospective cohort, and a genetic cohort study, they were well‐designed and had robust inclusion criteria along with a battery of index testing. Individualistic differences of index tests, patient populations, and evaluations are listed (Table 4). A proportion of the sample size presented mild hepatic WD, whereas neurological cases and asymptomatic WD presentations were also included.

**TABLE 4:** Characteristics of included studies

Author (Year)	Title	Study type	Sample characteristics	Index laboratory testing	Biases
Ferenci et al. (2005)^17^	Diagnostic value of quantitative hepatic copper determination in patients with Wilson's disease	Case control*	Individuals with WD, of neuropsychiatric, hepatic, or asymptomatic type, controls without WD and participants with other hepatic pathologies	Hepatic copper was measured by atomic absorption spectroscopy	Given the non‐randomized enrollment and observational nature of the study, potential biases may have existed
Lu et al. (2010)^16^	The reassessment of the diagnostic value of 24‐hour urinary copper excretion in children with Wilson's disease	Case control*	The individuals had unknown hepatic pathologies	24‐hour urinary copper was measured by ICP mass spectrometry	Non‐randomization, selection bias of the case‐control design may have led to bias
Mak et al. (2008)^15^	Diagnostic accuracy of serum ceruloplasmin in Wilson's disease: determination of sensitivity and specificity by ROC curve analysis among ATP7B‐genotyped subjects	Case control*	Individuals with WD of neuropsychiatric, hepatic, or asymptomatic type, groups with no diagnosis of hepatic or neurological deficit, and normal controls	Ceruloplasmin with nephelometry Beckman Coulter IMMAGE	The nature of clinical reference standards in addition to non‐randomization and low acceptability of results with small sample size may lead to the risk of bias
Merle et al. (2009)^14^	Serum ceruloplasmin oxidase activity is a sensitive and highly specific diagnostic marker for Wilson's disease	Case control*	Individuals with WD, neurological or hepatic, with alternative hepatic pathologies, or normal controls	Ceruloplasmin nephelometry Dade Behring	Sample selection, non‐randomization, and case‐control amplify the risk of bias
Nicastro et al. (2010)^11^	Re‐evaluation of the diagnostic criteria for Wilson's disease in children with mild liver disease	Case control*	Confirmed WD patients either with asymptomatic family screening or hepatic etiology, with alternative hepatic pathologies, or normal controls	Hepatic copper with flame absorption spectrophotometry; urine copper using flame absorption spectrophotometry; ceruloplasmin using radial immunodiffusion NOR‐Partigen Behring	Case‐control study type increases the risk of non‐randomization and less diverse sample set
Sezer et al. (2014)^12^	Is it necessary to re‐evaluate diagnostic criteria for Wilson's disease in children?	Case control*	Patients with hepatic WD, alternative hepatic pathologies, or normal controls	Ceruloplasmin by immunoturbidimetry Roche Modular; urine copper with atomic absorption spectrophotometry AA‐6701F Shimadzu; and hepatic copper by atomic absorption spectrophotometry AA‐6701F Shimadzu	Selection bias, non‐randomization bias may increase the risk of bias or confounders
Xu et al. (2018)^13^	The optimal threshold of serum ceruloplasmin in the diagnosis of Wilson's disease: A large hospital‐based study	Cross‐sectional	Patients that underwent ceruloplasmin analysis were eligible to be included, and the tests/records were noted in a hospital center	Beckman Coulter Immage; ceruloplasmin using nephelometry	The cross‐sectional study design leads to increase the risk of selection bias
Yang et al. (2015)^18^	Prospective evaluation of the diagnostic accuracy of hepatic copper content, as determined using the entire core of a liver biopsy sample	Prospective cohort	Patients with suspected hepatic WD, family member of people with confirmed WD, or those that had alternative hepatic pathologies	Hepatic copper using atomic absorption spectrophotometry Beijing Purkinje General Instruments	The study had the minimal risk of bias, and the population was most reflective of clinical practice
Zarina et al. (2019)^21^	Association of Variants in the CP, ATOX1, and COMMD1 genes with Wilson's disease symptoms in Latvia	Genetic prospective cohort	Patients with WD: asymptomatic, hepatic, neurological/psychiatric, and neurological/hepatic	Direct sequences of the ATOX1, COMMD1, and CP genes; direct DNA sequencing of the ATP7B gene	The study had the minimal risk of bias other than the relatively less sample size as compared to a large genetic cohort study

Note

*All patients were being tested for WD, and the Leipzig criteria were the standard of reference.

### 3.2 Ceruloplasmin index test

Five of the 9 studies evaluated the threshold of ceruloplasmin across 4,281 individuals, among which 541 had WD. The cutoffs were defined by the Leipzig criteria (0.1 g/L and 0.2 g/L; 0.19 g/L) (Mak et al., 2008, Nicastro et al.^11^ 2010, Sezer et al.^12^ 2014, Xu et al.^13^ 2018, Merle et al.^14^ 2009) (Table 1). In these five studies, the optimal cutoff for the ceruloplasmin index test was determined to be between 0.14 and 0.2 g/L. The studies were well‐designed, clearly defined WD, and the details of the laboratory cutoffs were conducted using receiver operating characteristic (ROC) curve analysis. It may be noted that ceruloplasmin levels are lower in the neonatal age group; however, the levels rise in women who are currently pregnant or taking oral contraception and those undergoing acute inflammation. Nicastro et al.^11^ (2010) and Sezer et al.^12^ (2014) conducted their studies in the pediatric population; Mak et al.^15^ (2008) and Xu et al.^13^ (2018) used a mixed pediatric and adult population, and Merle et al.^14^ (2009) only enrolled the adult population. Because ceruloplasmin is synthesized in the liver, the levels are lower in other causes of chronic liver disease, enteropathies, and nephrotic syndrome as compared to WD. Literature suggests that individuals undergoing chelation therapy have lower levels of ceruloplasmin levels; however, Merle et al.^14^ present differing results where 65% of the sample was on penicillamine therapy but the minute differences in assay technology may have led to this effect. It is imperative to account for the different methods of the analyte—ceruloplasmin, which may vary in terms of reference range, precision, and bias. Therefore, the cutoff values identified as 0.14–0.2 g/L serve as an essential method that is paramount when the same sample is run using the same analyte with a different method, but will possibly lead to different results as seen in the Merle study.^14^

### 3.3 24‐Hour urinary copper test

At present, there is limited evidence pertaining to the adult cutoff, with data enlisted by the European Association for the Study of the Liver (EASL) of 1.6 μmol/24 h. However, this systematic review presents three studies that enlist the 24‐hour urinary copper cutoffs of children with WD‐associated CLD (Lu et al.^16^ 2010, Nicastro et al.^11^ 2010, Sezer et al.^12^ 2014) (Table 2). Lu and colleagues utilized 24‐hour urinary copper was measured by ICP mass spectrometry using a Chinese population,^16^ whereas Nicastro/Sezer and et al.^11^, ^12^ used an atomic absorption spectroscopy in an Italian and Turkish population, respectively (Table 2). Both these studies used a clear criterion for diagnosing WD, and there were gender‐ and age‐matched controls. Wherever differences in cutoffs were present, they may have occurred due to slight differences in methods and ethnicity.

### 3.4 Hepatic copper

On considering the cutoffs for copper, four studies in total were eligible, and differences in age groups, methods, and index test analysis were presented (Table 3). Ferenci and colleagues clearly defined the WD criteria and employed a pediatric and adult population, and adequate laboratory methods were utilized to evaluate hepatic copper by using atomic absorption spectroscopy.^17^ The optimal cutoff based on the ROC curve analysis was 1.2 μmol/g, giving it a power of 96.4% sensitivity and 95.4% specificity. This is in opposition to the higher cutoff used in the Leipzig criteria of 4 μmol/g. However, the Leipzig criteria for hepatic copper were originally based on a sample of 7 individuals with WD as posited by Ferenci and colleagues.^17^ When noting the study by Yang and colleagues, which employed a Chinese sample set, the authors supported the 4 μmol/g cutoff, with 99.4% sensitivity and 96.1% specificity.^18^ Yang and authors had a larger sample size as compared to the former studies and clearly defined the criteria and methodology of the adult sample included.^18^ Minute differences may exist because distinct methodologies may also reflect ethnic and racial differences in alleles causing disease in the populations. Both Sezer and Nicastro tested hepatic copper using different methodologies of flame atomic absorption spectroscopy in the pediatric population with hepatic disease in Turkey and Italy, respectively.^11^, ^12^ While Nicastro did not undertake a ROC analysis, the authors used a 4 μmol/g cutoff based on the Leipzig criteria, yielding a sensitivity of 65% and specificity of 77%.^11^ On the contrary, Sezer noted that when the cutoff was decreased to a threshold of 1.5 μmol/g, the sensitivity increased to 91.4%, but the specificity decreased to 65.8%.^12^

## 4 DISCUSSION

This systematic review assesses the clinical, biochemical, immunological, and genetic tests that are included in the heterogeneous process of diagnosing WD. It may be stated that the variability in optimal cutoff values according to ROC curve analysis depends on the applied biochemical tests methodology and the age of participants as claimed by the evidence provided by the studies in this review. Notably, EASL guidelines supply a narrative expert‐base review about current evidence supporting the original Leipzig criteria. Cauza et al.^19^ conducted an assessment of 17 patients that had ceruloplasmin levels less than 20 mg/dl. While only one asymptomatic patient had WD with no neurological signs of Kayser‐Fleischer rings, the other 16 patients were considered to be heterozygous carriers of the WD gene. The positive predictive value of low ceruloplasmin was merely 5.9%. Despite considerations regarding the measurement of hepatic copper content as the gold standard for diagnosing WD, Ferenci and colleagues, in one of the largest studies in this area, highlight the occurrence of false‐negative results due to sampling errors or differences in hepatic copper distribution.^17^

Nine studies, including a total of 5,762 participants (which comprised of 1,163 individuals with Wilson's disease) were assessed. Three studies involved the pediatric population only, one comprised of adults only, and the other five studies involved both adult and pediatric populations. Two studies assessed individuals with hepatic signs only, and the other seven studies assessed the combination of neurological and hepatic signs and symptoms of WD. There were variable methodological qualities of the studies, the risk of bias due to the study types was documented, and the key differences in assays and cutoff values for the diagnostic tests are enlisted in Tables 1, 2, 3, 4.

When noting the ceruloplasmin index test, a total of five studies assessed the thresholds, pooling in 4,281 individuals with 541 confirmed WD patients. The all‐around scalable cutoff value was found to be ranging from 0.14 to 0.2 g/L. When using a cutoff of 0.2 g/L as per the Leipzig criteria, a sensitivity of 77.1%–99% was achieved and the specificity ranged from 55.9% to 82.8%. On noting the 0.1 g/L cutoff as per the Leipzig criteria, the sensitivity ranged from 65% to 78.9%, whereas the specificity ranged from 96.6% to 100%. The best practice guidelines resonate that a highly sensitive test leads to fewer false‐negative results, and fewer WD cases are missed, whereas the specificity of the ceruloplasmin test means that there are fewer false‐positive results. The most scalable test result has maximum sensitivity and specificity. While the current literature states that the ceruloplasmin level for WD will probably be below 0.1 g/L, our synthesis identifies,^20^ based on the data obtained by five studies, the optimal cutoff for the ceruloplasmin index test was determined to be between 0.14 and 0.2 g/L.

The 24‐hour urinary copper test was evaluated based on three studies, and there were varying thresholds using a participant size of 268, with 101 confirmed WD patients. While EASL posts 1.6 μmol/24 h as the cutoff, we found that the best practice evidence suggests a cutoff ranging from 0.64 to 1.6 μmol/24 h based on the Leipzig criteria, which achieves a sensitivity vs. specificity of 50% vs. 80% and 75.6% vs. 98.3%, respectively.

The hepatic copper test assessed a total of 1150 individuals with 367 WD patients. The hepatic cutoff in the Leipzig criteria achieved a sensitivity of 65.7%–94.4% and specificity of 52.2%–98.6%.

Overall, the cutoffs for ceruloplasmin, 24‐hour urinary copper, and hepatic copper test are reliant on the methodologies adopted by the testing facility and do require validation in the population when the index tests are used at large. While we provide cutoffs of the three tests for WD, we find that cutoffs are typically based on the specific subgroups based on ethnicity, age, and clinical subgroups.

While the findings of this systematic review may be used by the readers, and despite the included studies employing the Leipzig WD criteria, some of the included studies had limited details about the methodologies and the calculations, despite mentioning the index tests being appraised. While the current literature addresses the possible gold standards to diagnose WD, there is a lack of disease definition and there is a poorly defined index test methodology. Moreover, while ceruloplasmin levels are helpful in diagnosing WD, the feasibility and cost‐effectiveness of utilizing serum ceruloplasmin in presymptomatic WD may be low.^19^ With these limitations in mind, we believe that the methodological weaknesses of the included studies may have resulted in overestimation of the accuracy of the index tests, despite still offering pertinent information to medical communities worldwide.

## CONFLICT OF INTEREST

None.

## Data Availability

All data used and acquired for this study are available online.
